# PROTOCOL: Does executive compensation predict publicly traded firms’ financial performance or inaccurate financial reporting?

**DOI:** 10.1002/cl2.1064

**Published:** 2019-12-02

**Authors:** Denise M. Rousseau, Donna Beck, ByeongJo Kim, Ryan Splenda, Sarah Young

**Affiliations:** ^1^ Heinz College and Tepper School of Business Carnegie Mellon University Pittsburgh Pennsylvania; ^2^ University Libraries Carnegie Mellon University Pittsburgh Pennsylvania; ^3^ Heinz College Carnegie Mellon University Pittsburgh Pennsylvania; ^4^ University Libraries Carnegie Mellon University Pittsburgh Pennsylvania

## Abstract

This is the protocol for a Campbell review. The objectives are as follows: One goal of this systematic review is to identify whether incentive terms in CEO contracts predict firm financial performance over time; a second goal is to identify whether incentive terms in CEO contracts predict subsequent inaccurate financial reporting as manifest in restatement of accounting data due to errors or other distortions in reporting financial information.

## 1 BACKGROUND

### 1.1 Description of the condition

Chief Executive Officer (CEO) incentive compensation is intended to motivate chief executives to help their firms attain important business goals by aligning CEO interests with those of the firm's stakeholders, including investors, employees, and others. Targets may be short‐term such as an annual increase in stock price, or long‐term such as revenue growth over a period of years. Such financial performance metrics are typical performance targets established in CEO incentive compensation. They provide evidence of the firm's financial well‐being and future prognosis of interest to the firm's current stockholders and future investors. However, such metrics are compiled by a firm's employees and are potentially subject to inaccurate reporting. This inaccurate reporting is attributable at times to errors and other times to bias in the information reported as a result of direct and indirect influence by the CEO and that person's direct reports, which can result in fraud. Financial restatements are changes in reports of business outcomes due to inaccuracies or errors identified through audits by company accountants or outside auditors.

### 1.2 Description of the intervention

Incentive compensation refers to formal contracts to provide a bonus or pay increase contingent on the firm attaining a performance target or targets—a common feature of publicly traded firms. A recent report by the compensation research firm Equilar compiled data reflecting pay for the chief executives at 199 public companies, indicating that over 95% had incentive compensation (the remaining handful of firms had CEOs with substantial ownership in the firm; Pay at the top, [Link]). Incentive compensation for meeting performance targets like total revenue, change in net income or change in shareholder return can include cash bonuses (Ashley & Yang, 2004; Coleman, 2000; Nourayi & Mintz, 2008) as well as equity compensation (e.g., stock options; Ashley & Yang, 2004; Jeppson, Smith, & Stone, 2009).

### 1.3 How the intervention might work

Incentives are expected to direct CEO attention to certain outcomes and away from others. Moreover, since CEOs can negotiate their own contracts, they may bargain for terms they believe can be more readily attained. Incentives also can motivate CEOs to direct the activities of their subordinate managers and others in order to realize the financial performance outcomes specified in the incentive contract. A by‐product of this incentivized attention to certain outcomes can be inaccurate financial reporting due to manipulation of the firm's financial data so that the CEO receives the contracted incentives. Such manipulation is one cause of the need to correct or restate previously filed financial information regarding the firm. The regulatory environment may influence the incidence of financial restatement, and any link with financial incentives: Recent research conducted in the aftermath of the Sarbanes‐Oxley Act of 2002, which made broad changes in financial reporting requirements, suggests that efforts at deterrence and detection of misstatements have been successful, with a small portion of the restatements by public companies in recent years judged to be fraudulent (Alali & Wang, 2017).

### 1.4 Why it is important to do this review

This is the first systematic review on the topic of CEO incentive compensation and it comes at a time when CEO pay in the United States has risen 940% since 1978 relative to a 12% increase for rank and file employees (Mishel & Wolfe, 2019). The incentive compensation of CEOs is a major organizational decision, with implications for stakeholders, the firm, and the broader society. For governing boards of corporations, CEO pay is a major decision with potential implications for firm performance, effective use of resources, employee well‐being, and long‐term organizational consequences. Since CEOs negotiate their own contracts, they may bargain for terms more favorable to themselves than to the firm's other stakeholders. Moreover, incentives can have unintended consequences including the manipulation of accounting data in order to increase the likelihood of receiving contracted incentives. Disputes regarding the efficacy of CEO incentive compensation involve whether CEOs are paid too much relative to the salaries of rank and file employees or their contribution to the firm's success.

## 2 OBJECTIVES

One goal of this systematic review is to identify whether incentive terms in CEO contracts predict firm financial performance over time; a second goal is to identify whether incentive terms in CEO contracts predict subsequent inaccurate financial reporting as manifested in restatement of accounting data due to errors or other distortions in reporting financial information.

## 3 METHODS

### 3.1 Criteria for considering studies for this review

#### 3.1.1 Types of studies

Because we focus on the prediction of firm performance and financial restatements by CEO financial incentives, this review requires evidence that predictors (incentives) occur before outcomes. Eligible studies are longitudinal in nature with financial outcomes and/or financial restatement measured at a later point in time than the incentive measures. Included studies will use controls for (a) preincentive firm performance and/or (b) market conditions prevailing at the time longitudinal firm performance measures are gathered (e.g., random effects [luck] that can increase market‐related outcomes, such as increase in oil price for firms in the petroleum industry, Bertrand & Mullainathan, 2001). Eligible studies include those where CEO financial incentives serve as predictors for outcomes measured later and may include comparison groups where incentives differ.

#### 3.1.2 Types of participants

Included studies will be limited to those that focus on publicly traded firms. Studies that focus on private companies will be excluded. Included studies will be limited to those examining the incentive contracts of CEOs. (We will exclude data from other organizational executives such as Chief Operating Officers and the like.)

#### 3.1.3 Types of interventions

Incentive contracts include all formal agreements entered into between a corporate board and the CEO of a publicly traded company in which future rewards are offered contingent on attaining specified levels of performance or performance targets. Such contracts can include cash bonuses, stock options, and other financial instruments offered based on the attainment of future performance outcomes. (Note incentives are distinct from prespecified salary levels but can include salary increases specified in advance for the attainment of performance targets.)

N.B. Randomized controlled studies are unlikely in this context.

#### 3.1.4 Types of outcome measures

##### Primary outcomes

Our first primary outcome is firm financial performance. We will identify the financial outcomes studies report categorizing them according to their time frame (1, 2–3, 4+ years) and type of performance, that is, (a) profitability indicators including return on investment (ROI), return on assets (ROA), and other standard profitability metrics and (b) market returns including changes in market‐to‐book value and other indicators of increased shareholder returns. We thus expect to record the time lag reflected in each financial outcome and to analyze outcome data as a function of their time lag, that is, for example, grouping ROI measures at Year 1 together, ROI measures at Year 2 together, and so forth.

Our second primary outcome is whether financial restatements have been made. Financial restatements are corrections to previously issued accounting results for the firm and are used as an indicator of manipulation or misspecification of outcomes attained during a CEO's tenure.

All outcomes will be derived from archival data as reported in studies included in this review. We will include both studies with useable data and those whose data are ultimately deemed unuseable for constructing effect sizes. For the latter, we intend to seek information from authors when needed to increase the usability of their data.

##### Secondary outcomes

None

### 3.2 Search methods for identification of studies

To address the question of the effect of executive compensation on publicly traded firms' financial performance and financial restatements, we developed a search strategy that will help focus our search of the overall compensation literature. Our search strategy aims to limit results to the following:

- Research reported since January 1, 1980 (this time frame is used to anchor findings in the economic era that began with Margaret Thatcher becoming prime minister in the UK in 1979 and Ronald Reagan President of the United States in 1981 and concomitant changes in tax structure and corporate regulation initiated and sustained since then).
- Research focused on CEO compensation.
- Research that measures publicly traded firms' financial performance and/or financial restatements.

The search strategy will be used in a number of electronic search outlets yielding a comprehensive corpus of executive compensation research. These outlets will include subject‐specific and multidisciplinary bibliographic databases and subject‐specific gray literature websites and repositories.

In the bibliographic databases, we will use a combination of subject/thesaurus terms and keywords to find relevant executive compensation literature. Searches will be limited to titles and abstracts. For the gray literature websites and repositories, advanced keyword searching will be used if available. The subject terms and keywords will address CEO compensation, firm financial performance, and financial misreporting.

A hand search of relevant journals in which studies on this topic tend to be found, but that are not indexed in the bibliographic databases, will be performed to identify additional primary studies.

Additional relevant studies will be harvested from the references of the studies identified for inclusion, as well as related literature reviews. Titles of included studies will be searched in Google Scholar and references citing those titles will be reviewed for inclusion.

#### 3.2.1 Electronic searches

The primary search outlets that will be used to gather relevant studies are the bibliographic databases. The databases that will be included in our search are as follows:

- ABI/INFORM (ProQuest)
- Business Source Ultimate (EBSCO)
- Emerald Management Research Collection
- EconLit (EBSCO)
- Web of Science Core Collection
- Scopus
- Dissertations and Theses (ProQuest)
- Directory of Open Access Journals (DOAJ)

A search strategy developed for ABI/INFORM is included in the Supporting Information. This search will be translated across the abovementioned database platforms. Searches will be limited to articles reported from January 1, 1980, onward. Additionally, in ABI/INFORM and Business Source Ultimate, we will use available filters to remove news articles, trade journals, industry reports, and magazine articles.

#### 3.2.2 Searching other resources

##### Gray literature

In addition to the bibliographic databases, we will search a number of gray literature resources, including conference proceedings and papers not indexed in the electronic databases, working papers, white papers, and other types of information from January 1, 1980, onward. Basic keyword searches with terms related to CEO compensation, firm financial performance, and financial restatements will be carried out for the following websites:

- National Bureau of Economic Research (NBER) Working Papers: https://www.nber.org/papers.html
- Bureau of Economic Analysis (BEA): https://www.bea.gov/
- Board of Governors of the Federal Reserve System publications: https://www.federalreserve.gov/publications.htm
- Federal Reserve Economic Data (FRED): https://fred.stlouisfed.org/
- 2018 American Economic Association (AEA) Papers & Proceedings journal (previous years indexed in Business Source Ultimate)
- Social Sciences Research Network (SSRN): https://www.ssrn.com/en/
- Research Papers in Economics (RePEc) IDEAS: https://ideas.repec.org/
- Centre for Economic Policy Research https://cepr.org/
- Business Council of Canada https://thebusinesscouncil.ca/
- Conference Board ‐ Business Management Research: https://www.conference‐board.org/ea/search.cfm

When advanced or structured searching mechanisms exist within these websites, we will include detailed search strategies for those cases. Gray literature reported prior to January 1, 1980 will be excluded and a date limit will be applied to the search when possible.

##### Hand searching

Some hand searching will supplement the electronic database searching. We will screen tables of contents and reference sections in the following journals for additional relevant studies:

1. *Advances in Business Research*: http://journals.sfu.ca/abr/index.php/abr/ (from its inception in 2010 to present day)
2. Academy of Management Review (past 1 year)
3. Academy of Management Annals (past 1 year)

A number of management conferences produce conference proceeding reports or publications. Many of these are indexed in the abovementioned electronic bibliographic databases (e.g., the Academy of Management Proceedings in Business Source Ultimate). The following conference proceedings/publications are not indexed in databases and will be screened for relevant studies on their openly available websites, including only research since January 1980:

- International Conference on Economics, Business and Management (ICEBM)—Journal of Economics, Business and Management: http://www.joebm.com/list‐6‐1.html
- International Conference on Advances in Management Sciences (ICAMS)—Journal of Advanced Management Science: http://www.joams.com/index.php?m=content&c=index&a=lists&catid=9
- Academy of International Business (AIB)—Proceedings of the Annual Meeting of the Academy of International Business: https://aib.msu.edu/publications/confproceed.asp

We will screen the references of the included studies, as well as those in related literature reviews published after January 1980. Titles of included studies will also be searched in Google Scholar and references citing those titles will be reviewed for inclusion.

Finally, we will contact selected subject matter experts to determine whether there are additional in press or unpublished studies relevant to our questions.

### 3.3 Data collection and analysis

#### 3.3.1 Selection of studies

All results from database and gray literature searches will be added to Covidence or a similar program, which will be used to manage the process of deduplication and study screening. After all, searches are conducted and the deduplication process is complete, two of the review authors will independently screen all titles and abstracts, excluding studies that are clearly irrelevant to the review question. Any studies that are deemed to possibly meet inclusion criteria by at least one reviewer, or for which there is insufficient information to determine eligibility, will be retrieved in full text. Two authors will then independently review the full text of these studies to determine eligibility based on the criteria outlined in the Supporting Information. Any disagreements between reviewers will be resolved by discussion and consensus. Studies excluded at this stage will be assigned a reason for exclusion.

The eligibility criteria will be piloted by the reviewers on a total of 10 studies and clarifications made to ensure that the criteria are correctly interpreted and applied by all reviewers.

#### 3.3.2 Data extraction and management

Two review authors will independently code and extract desired data from each of the included studies using the data extraction form in the Supporting Information. The data extraction form will be piloted on a small number of studies and revised as needed. Disagreements between reviewers will be resolved through discussion and consensus, and subject matter experts consulted when necessary.

If important data are missing, we will contact the authors of identified studies in order to obtain more complete information.

#### 3.3.3 Assessment of risk of bias in included studies

Multiple raters will assess the study‐level risk of bias after primary studies are identified and full‐text has been extracted. The risk of bias will be assessed in terms of sample representativeness (of the population) and missing data. The clarity and reliability of performance measure reporting will also be evaluated. In addition, we will evaluate the appropriateness of controls used in analyses.

#### 3.3.4 Measures of treatment effect

We will use "r" as our indicator of treatment effect since our studies will typically report findings as regression coefficients computed for data that are continuous and observational. If reports are incomplete with respect to effect size indicators we will consult sources (e.g., Lipsey et al., 2012) for other equations that can calculate such estimates. Campbell methodologists will be consulted on the synthesis of different correlation metrics.

#### 3.3.5 Unit of analysis issues

We will focus on organization‐level studies to assess CEO effects in terms of organization‐wide outcomes as specified in CEO contracts. In studies where dependent effects might exist, we will follow Cochrane Handbook protocols by separating out analyses of specific effects by type of outcome (e.g., ROI, stock price) or time lag (1, 2 years, etc.). Since firms have only one CEO, included studies will not need adjustments for clustered data.

#### 3.3.6 Dealing with missing data

Incomplete information about studies will be sought by contacting authors and searching for additional reports of those studies. We will consult our advisory team regarding appropriate strategies for dealing with missing data.

#### 3.3.7 Assessment of heterogeneity

Some substantive differences may exist including time periods studied (1980s–2010s), firm size and country context. Methodological differences including analytic methods (regression vs. bivariate analyses), differences in covariates and other factors related to the risk of bias may also be factors and will be coded to assess the effects of heterogeneity. We plan to use several indicators including REVMAN's Chi‐square test for heterogeneity and the Tau‐squared statistic. We will also examine the forest plot to see if confidence intervals for studies' effect size overlap.

#### 3.3.8 Assessment of reporting biases

If we have more than 10 primary studies, as we anticipate, we will consult a Campbell methodologist in order to investigate publication bias.

#### 3.3.9 Data synthesis

We will use REVMAN for random‐effects meta‐analysis, using the "r" metric for our effect size indicator. Random effects models will be used as it is unlikely that all studies produce estimates of a single population parameter. We will use inverse variance methods to weight study effect sizes by their precision in our meta‐analysis. We will use STATA to test for moderator effects.

Descriptive statistics will be provided on the set of studies included in our review. These indicators will include means and standard deviations for variables characterizing the studies (including sample size, CEO tenure/age, frequency and percentages of country, industry, and other qualitative characteristics, etc.).

#### 3.3.10 Subgroup analysis and investigation of heterogeneity

We will examine whether risk of bias levels affect effect sizes along with potential effects attributable to time frame, firm size, country context, and methodological differences (e.g., analytic method, and the number and type of covariates). In addition, we will examine the potential moderating effect of past firm performance during the CEO's tenure and the CEO's chronological age. The logic for these moderation analyses, consistent with some current literature findings, is as follows: (a) a board's prior experience with a CEO including the level of previous performance attained under that CEO is expected to affect the nature of incentives offered and (b) the CEO's age is expected to be associated with different preferences for pay at risk (performance incentives) versus salary (Al Shammari, 2018; McClelland, Barker, & Oh, 2012). Past firm performance refers to levels of firm performance prior to or at the time the CEO's financial incentive contract is created and will serve as a control in order to assess the degree of change in firm performance predicted by CEO financial incentives.

Metareg in STATA can be used to test moderator effects.

#### 3.3.11 Sensitivity analysis

Sensitivity analysis will be conducted to address any deviations from the protocol made as a result of our review and analysis of the literature (e.g., changes in inclusion criteria).

Note we have not yet developed a summary table for our findings given the potential array of financial contract terms and outcome indicators relevant to our questions. Such a table will be developed after we have completed the initial phases of our review.

## CONFLICT OF INTERESTS

The authors declare that there are no conflict of interests.

## AUTHOR CONTRIBUTION

D. R., an organizational psychologist, is primarily responsible for writing the protocol. Responsibilities include formulating the overview of the project, interpreting relevant literature in problem framing, and assisting in identifying relevant concepts to construct our PICO. Our team librarians, D. B., R. S., and S. Y. developed the search strategy and identified appropriate search terms. R. S., a business librarian, also contributed to the formulation of the outcome measures appropriate for addressing our question. S. Y., contributed particularly to development of our extraction documentation. B. K., a doctoral student, assisted in literature reviews as we prepared our specific search strategy.

## Supporting information

Supplementary information
