# Supplementary material for: PROTOCOL: Does executive compensation predict publicly traded firms’ financial performance or inaccurate financial reporting?
Source: Campbell Syst Rev. 2019 Dec 2;15(4):e1064. doi: 10.1002/cl2.1064 (PMC8356531; doi:10.1002/cl2.1064)
Supplement: Supplementary file 1 — Supplementary information [file CL2-15-e1064-s001.docx]

# Appendices

## 1 ABI/INFORM search strategy

| 1 | MAINSUBJECT("Chief executive officer*") |
| --- | --- |
| 2 | AB,TI(ceo OR ceos OR "chief executive officer*" OR "senior leader*" OR “corporate director*” OR executive*) |
| 3 | 1 OR 2 |
| 4 | MAINSUBJECT(Bonus* OR “monetary incentive*” OR “productivity incentive*” OR compensat* OR “compensat* plans” OR “incentive plan*” OR “pay for performance” OR “unreasonable compensation” OR “stock option*” OR “executive compensation”) |
| 5 | AB,TI(bonus* OR incentiv* OR “pay for performance” OR compensation OR salary OR salaries OR “stock options” OR “performance award*” OR “performance based pay” OR “performance pay” OR “performance-related pay” OR “executive pay” OR “executive profit sharing”) |
| 6 | 4 OR 5 |
| 7 | MAINSUBJECT.EXACT(“financial performance” OR “corporate profits” OR earnings OR “retained earnings” OR “undistributed profits” OR “earnings per share” OR “return on investment” OR revenue OR “financial ratios” OR “return on assets” OR “return on equity” OR “corporate mergers” OR "market value") |
| 8 | AB,TI(firm* OR corporate OR corporation* OR company OR companies OR organizational OR financial OR business) NEAR/5 AB,TI(performance OR profit* OR “stock price” OR value OR sales OR revenue OR “market share” OR innovation OR growth OR “financial health” OR liquidity) |
| 9 | AB,TI(“return on assets” OR ROA OR “return on income” OR ROI OR ROIC OR “return on invested capital” OR “return on capital” OR ROC OR “return on equity” OR ROE OR EBITDA OR “earnings before interest tax depreciation and amortization” OR “stock market returns” OR “equity pricing” OR “profit margin” OR “financial ratio” OR “merger and acquisition” OR “mergers and acquisitions” OR "market value" OR "market return*" OR "market to book" OR "price to book" OR "total shareholder return") |
| 10 | 7 OR 8 OR 9 |
| 11 | MAINSUBJECT.EXACT(“financial restatements” OR “accounting irregularities”) |
| 12 | AB,TI(financ* OR account* OR corporate) NEAR/5 AB,TI(misrepresent* OR restatement* OR mislead* OR negligen* OR irregular* OR inaccura* OR inconsisten* OR manipulate* OR decept* OR dishonest OR deceive* OR misreport* OR fraud* OR lying OR false) |
| 13 | 11 OR 12 |
| 14 | 10 OR 13 |
| 15 | 3 AND 6 AND 14 |
| 16 | Limits: Source Type—Scholarly Journals, Dissertations & Theses, Working Papers, Conference Papers & Proceedings |
| 17 | Limit: 1980-01-01 to present |

## 2 Data Extraction Form

The following fields will be used to code and extract data from each article. All data will be initially coded in Excel for tracking purposes and warehousing information.

**I. Relevance Screening**

a.Document Full Citation (APA style)

b. Is the document about a longitudinal study?
⎕ Yes
⎕ Unclear
⎕ No (explain) – STOP REVIEW

c. Timing: Was the study reported post January 1980?
⎕ Yes (initial year of data collection)?
⎕ No (explain) – STOP REVIEW

d. Does the study examine CEO incentive contracts?
⎕ Yes
⎕ No – STOP REVIEW

e. Does the study include a metric of firm performance or financial restatements measured after CEO incentive contracts?
⎕ Yes
⎕ No – STOP REVIEW

f. Does the study include publically traded corporations?
⎕ Yes
⎕ No – STOP REVIEW

g. Does the study include controls for a) pre-incentive firm performance and/or b) market conditions prevailing at the time?

⎕ Yes
⎕ No – STOP REVIEW

II. **Data Extraction Worksheet**

**Study ID:**

**Coder Name:**

**Study description**

a. How many documents are associated with this study?

b. Type of publication [for each study document]
⎕ Book
⎕ Peer-reviewed journal article
⎕ Book chapter (in an edited book)
⎕ Thesis or dissertation
⎕ Technical report
⎕ Conference paper
⎕ Other:___________________
⎕ Unreported/cannot tell

c. Countries in which study was conducted [check all that apply]
⎕ USA
⎕ Canada
⎕ Great Britain
⎕ Other English speaking
⎕ Other ____________________
⎕ Unreported/cannot tell

d. Industries in which study was conducted
⎕ Diverse industries of publicly traded firms
⎕ Industry subset of publicly traded firms? Which?____________

e. Sample size - number of firms included in the study

f. Funding sources, if any (for the study)

**Study methods**

a. Were comparison groups included?

⎕ Yes [explain nature of comparisons, e.g., CEO incentive versus no incentive, or comparison of different types of incentives]

⎕ No

**Firm characteristics**

a. number of employees (minimum, maximum, mean, standard deviation)

b. number of sites/locations per firm (min, max, mean, sd)

c. annual revenue (min, max, mean, sd)

**CEO characteristics**

a, age (min, max, mean, sd)

b. tenure [years in position] (min, max, mean, sd)

c, gender [% in subgroups]

d. race/ethcnicity [% in subgroups]

e. base salary (min, max, mean, sd)

**CEO incentive schemes**

a. What types of incentives were studied [check all that apply]

⎕ Cash bonus

⎕ Stock options

⎕ Salary increase

⎕ Other [explain]

b. What targets were tied to incentives [check all that apply]

⎕ Stock price

⎕ Revenue growth

⎕ Other [explain]

**Firm performance measures**

a. What performance measures were included? [check all that apply; for each, explain how the construct was operationalized and how [and by whom] measures were created/obtained}

⎕ Profitability: Return on investment (ROI)

⎕ Profitability: Return on Assets (ROA)

⎕ Profitability: Return on Assets (ROIC)

⎕ Profitability: Return on Assets (ROC)

⎕ Profitability: Return on Assets (ROE)

⎕ Profitability: Return on Assets (EBITDA)

⎕ Market returns: Market-to-book value

⎕ Other indicators of increased shareholder returns

⎕ Other [explain]

⎕ None

b. Timing of performance measures available [check all that apply]

⎕ prior to incentive contract

⎕ at the time of the incentive contract

⎕ within one year after the contract

⎕ >1-2 years after

⎕ >2-3 years

⎕ >4-5 years

⎕ more than 5 years

⎕ Not applicable

**Financial restatement measures**

a. Are financial restatement indicators available?

⎕ Yes - explain how [and by whom] restatement indicators were created/obtained

⎕ No

**Analytic methods**

a. Statistical methods used [check all that apply]

⎕ bivariate associations

⎕ multivariate models - identify types of models used

⎕ other [explain]

b. Statistics reported [check all that apply]

⎕ correlation coefficients (unadjusted)

⎕ partial correlations

⎕ regression coefficients

⎕ other [explain]

c. Covariates used in regression (or other multivariate) models [number and type of covariates]

d. Was data imputation used to fill in missing data?

⎕ Yes: Describe imputation methods used _____________

⎕ No

⎕ Unclear

**Results**

a. For each relevant analysis in the study, we will extract data on

⎕ type of CEO incentive and target

⎕ nature and timing of outcome measures (performance indicators or financial restatement)

⎕ valid n

⎕ type of effect size [r, partial r, b or other]

⎕ values of effect sizes

⎕ values of SE or other measures of variability [e.g., confidence interval]

⎕ number and types of covariates in the model, if any

⎕ p-values

⎕ method of imputation, if any

**III. Risk of Bias Assessment**

a. Selection bias: Nonequivalent comparison groups

⎕ Low risk [comparison groups were equivalent at baseline, i.e., no initial differences between CEOs with and without incentive contracts]

⎕ High risk [comparison groups exhibited differences at baseline]

⎕ Unclear

⎕ Not applicable [comparison groups were not used]

b. Selection bias: Representativeness

⎕ Low risk [representative sample of larger population of firms]

⎕ High risk [convenience sample of firms]

⎕ Unclear

c. Missing data: Attrition bias

⎕ Low risk [results for all firms are fully reported]

⎕ High risk [incomplete outcome data for some or all firms, e.g., missing data on financial restatements]

⎕ Unclear

d.Missing data: Outcome reporting bias

⎕ Low risk [outcomes are fully reported regardless of direction and significance of results]

⎕ High risk [statsitically non-significant results are not fully reported, e.g., missing r or SE or valid n]

⎕ Unclear

e. Detection bias: Unreliability of measures

⎕ Low risk [all measures are judged to be reliable and valid]

⎕ High risk [some or all outcome measures appear to be unreliable or invalid]

⎕ Unclear

f. Other sources of bias: Lack of adequate controls for relevant confounders

⎕ Low risk [relevant confounders are included as controls in analysis]

⎕ High risk

⎕ Unclear
